# Job shadowing adults' non-formal education programme through social-emotional learning

**DOI:** 10.3389/fsoc.2025.1524922

**Published:** 2025-08-26

**Authors:** Ieva Margeviča-Grinberga, Aija Kalēja

**Affiliations:** Faculty of Education Sciences and Psychology, University of Latvia, Riga, Latvia

**Keywords:** job shadowing, non-formal education, social-emotional learning, social skills, participant satisfaction, professional identity

## Abstract

This study explores the impact of a job shadowing non-formal education program, integrated with social and emotional learning (SEL), on adult professional development and career planning. The program engaged adults from diverse professional backgrounds, providing them with structured opportunities to observe and interact with experienced professionals in real workplace environments. A mixed-methods approach was employed, combining self-assessment questionnaires, interviews, and reflective journals to capture both quantitative and qualitative dimensions of participant experience. Findings indicate that participation in SEL-based job shadowing fosters the development of professional identity, emotional intelligence, and adaptability among adult learners. Participants reported increased self-confidence, enhanced workplace communication, and greater clarity in career direction. The program also supported the acquisition of practical skills and encouraged ongoing professional learning, with many participants expressing motivation to pursue further education or training. The results highlight the value of integrating SEL principles into job shadowing as a non-formal education programme, demonstrating its effectiveness in supporting career transitions, lifelong learning, and workforce readiness in a rapidly changing labor market. These insights underscore the importance of designing job shadowing as a non-formal education programme that not only addresses skill development but also promotes emotional and social competencies essential for sustainable professional growth. This study contributes to the theoretical understanding of how experiential, SEL-driven job shadowing can facilitate holistic adult development and provides practical recommendations for educators, policymakers, and employers seeking to enhance the relevance and impact of non-formal learning programs for adults.

## 1 Introduction

In the context of rapid changes in the labor market and increasing demands for adaptability, adult learners face the challenge of acquiring not only new skills but also relevant professional experience to support successful career transitions. The ability to make informed career choices and develop competencies that align with evolving workplace requirements is a critical issue in adult education and lifelong learning (Ball, 2021). Job shadowing, defined as a structured opportunity for individuals to observe professionals in real work environments, has emerged as a promising method for bridging the gap between theoretical knowledge and practical experience (Czarniawska, 2007; Bickerton, 2023).

Despite its recognized potential, job shadowing remains underutilized and inconsistently implemented in adult education, often limited by organizational constraints and a lack of structured programs (Valko-Juhasz, 2022). Yet, research indicates that job shadowing can facilitate career exploration, increase motivation, and provide authentic exposure to diverse professional roles (Danijela, 2021; Mafinejad et al., 2022a). Holland's theory of vocational choice (Holland, 1997) underpins much of the rationale for job shadowing, positing that individuals are more satisfied and successful when their work environment aligns with their personality type. Job shadowing enables adults to test this fit in practice, supporting more conscious and sustainable career decisions (Adlya and Zola, 2022).

However, the effectiveness of job shadowing is significantly enhanced when integrated with social and emotional learning (SEL) principles. SEL focuses on the development of self-awareness, emotional intelligence, relationship skills, and responsible decision-making, competencies that are increasingly recognized as essential for success in modern workplaces (Goodman, 2024; Hildmann et al., 2019). Studies have shown that emotional intelligence is a critical factor in professional environments, influencing job satisfaction, teamwork, and adaptability (Zeidner et al., 2004; Joseph and Newman, 2010). Integrating SEL into job shadowing programs provides adult learners with not only technical and practical skills but also the emotional and social capacities needed to navigate complex career pathways and organizational cultures (Váradi, 2022; Wood, 2020).

This study is motivated by the need to address the scarcity of structured, SEL-based job shadowing as a non-formal education programme for adults and to evaluate its impact on professional development and career planning. By focusing on the intersection of job shadowing as a non-formal education programme and SEL, the research aims to provide evidence on how such programmes foster professional identity, emotional intelligence, and adaptability, ultimately enhancing employability and lifelong learning in a dynamic labor market.

The following sections will review the theoretical background, present the research methodology, and discuss the findings in relation to existing literature and practical implications for job shadowing as a non-formal education programme and workforce development.

## 2 Literature review

### 2.1 Background effects

The concept of “background effects” in this study refers to how an individual's personal, educational, and socio-economic background influences their engagement with and outcomes from job shadowing as a non-formal education programme. Background effects include factors such as prior work experience, educational attainment, personality traits, socio-economic status, and access to social support. These factors work together to shape a person's readiness for career development, their ability to adapt to new professional environments, and their responsiveness to job shadowing as a non-formal education programme.

A comprehensive theoretical framework for understanding these effects is provided by Holland's theory of vocational choice (Holland, 1997). Holland's model suggests that career satisfaction and success are greatest when there is alignment between an individual's personality type and their work environment. The theory describes six main personality types—Realistic, Investigative, Artistic, Social, Enterprising, and Conventional—each connected to particular occupational environments. According to Holland, both personal characteristics and environmental factors interact to influence vocational behavior and decision-making. Individuals receive a Holland code that reflects their interests and suitability for various occupations, which can then be used to guide career choices and educational pathways (Jigau, 2007).

Within job shadowing, background effects are visible in several ways. Participants' previous experiences and qualifications influence how they perceive and benefit from job shadowing opportunities. Those with relevant backgrounds may adapt more quickly and gain greater value from the experience. The fit between personality and environment, as emphasized by Holland's theory, is also important. Job shadowing programs that take into account participants' RIASEC profiles can increase satisfaction, engagement, and skill acquisition. Socio-economic and social factors, such as access to resources, social capital, and support networks, also play a role in determining participation and outcomes. Individuals from more advantaged backgrounds may have greater access to high-quality shadowing experiences and mentorship. Emotional intelligence, motivation, and self-efficacy, key components of social-emotional learning, mediate how individuals navigate new environments, interact with mentors, and manage the challenges of career transitions (Zeidner et al., 2004; Joseph and Newman, 2010).

By combining Holland's theoretical framework with an understanding of these background effects, job shadowing programs can be designed to better address the diverse needs of adult learners. This approach supports informed career decision-making and encourages the development of practical skills, emotional resilience, and professional identity.

In summary, background effects in this study are defined as the combined influence of personal, educational, and socio-economic factors that shape an individual's engagement with and outcomes from job shadowing as a non-formal education programme. Recognizing and addressing these effects is essential for maximizing the effectiveness of job shadowing as a non-formal education programme and supporting sustainable career development for adults.

### 2.2 Social and emotional learning in the context of job shadowing

Holland's theory of vocational choice not only addresses the compatibility between personality types and work environments but also highlights the critical role of social-emotional dimensions in professional development (Nicolini and Korica, 2024; Dalgic, 2017). Emotional intelligence, defined as the ability to perceive, understand, and regulate emotions, is widely recognized as a key workplace competency, closely linked to improved career satisfaction, adaptability, and performance (Zeidner et al., 2004; Joseph and Newman, 2010). Within job shadowing contexts, Holland's framework encourages meaningful social interaction between shadowers and professionals, thereby fostering a supportive and collaborative learning environment. Such environments are instrumental in enhancing participants' motivation, self-confidence, and capacity for informed career decision-making (Jigau, 2007).

Integrating social and emotional learning (SEL) principles into job shadowing as a non-formal education programme enables participants to develop essential competencies, including self-awareness, emotional regulation, empathy, and effective interpersonal communication. These skills are crucial for navigating new professional contexts, managing workplace challenges, and building a resilient career identity (Breanna, 2023; Huang et al., 2024). Research consistently shows that SEL competencies contribute to higher job satisfaction, greater career clarity, and enhanced adaptability to evolving labor market demands (Zeidner et al., 2004; Joseph and Newman, 2010).

Holland's theory further supports the development of these social-emotional skills by promoting the alignment of individual personality types with suitable work environments. This alignment not only helps individuals understand their interests and suitability for various occupations but also lays the foundation for the growth of social-emotional competencies essential for sustained professional development and lifelong learning (Holland, 1997; Adlya and Zola, 2022).

Table 1 illustrates how Holland's theoretical framework informs participant selection and career outcomes within job shadowing as a non-formal education programme. The table demonstrates the connections between personality types, career alignment, and social-emotional learning (SEL), categorizing the key elements of Holland's vocational theory. This approach clarifies how these principles shape both the structure of job shadowing as a non-formal education programme and the experiences of participants as they navigate career decisions.

**Table 1:** Influence of Holland's method on participant selection and career outcomes (Holland, 1966).

**Aspect**	**Impact**	**Social and emotional aspect**	**Source**
Job shadowing informal education programme	Provides clear guidelines and goals to improve program quality and accessibility	Promotes emotional intelligence and social skills, increasing confidence and motivation	Holland, 1966; Goodman, 2024
Compatibility of personality and working environment	Matches personality type with career selection	Helps individuals build confidence in career decisions	Holland, 1966; Adlya and Zola, 2022
The Holland theory	Encourages thoughtful career choices	Supports informed career choices	Holland, 1966, 1997
Holland code	Assigns a three-letter personality code to individuals	Guides career exploration with hands-on experience	Holland, 1966, 1997; Zainudin et al., 2024
Hands-on experience	Gain real-world insight into occupations that help them make informed career decisions	Understand their strengths and weaknesses, which influence their career path	Frost, 2016; Margeviča-Grinberga and Kalēja, 2024
Career decision-making	Encourages thoughtful career choices	Emotional preparation through shadowing builds confidence	Holland, 1966; Hildmann et al., 2019

Data derived from Holland (1966) and supplemented with career development models from contemporary work-based learning research.

By grounding job shadowing as a non-formal education programme in both Holland's theory and SEL principles, educators and programme designers can create more effective, inclusive, and adaptive learning environments. Such environments support adult learners' professional growth and social-emotional wellbeing, ensuring that participants develop not only technical skills but also the personal and interpersonal competencies essential for sustainable career development (Goodman, 2024; Hildmann et al., 2019).

Holland's theory serves as a foundational framework for participant selection in job shadowing as a non-formal education programme (Holland, 1997; Adlya and Zola, 2022; Zainudin et al., 2024). By systematically assessing the compatibility between individual personality types and specific work environments, this theory enables the creation of more tailored and effective shadowing experiences. Such alignment not only enhances participant satisfaction and engagement, but also fosters more informed and responsible career decisions. Through this process, individuals benefit from both practical experience and the development of emotional readiness, which are essential for successful career exploration and adaptation.

This approach is supported by research demonstrating that when participants' vocational interests and personality profiles are matched with appropriate occupational environments, they experience greater job satisfaction, stability, and professional growth. The use of tools such as the Self-Directed Search (SDS) and RIASEC codes allows programme designers to identify optimal placements, ensuring that job shadowing as a non-formal education programme maximizes its developmental impact for each participant.

Beyond personality-environment fit, the psychological contexts and social-emotional aspects relevant to job shadowers encompass a wide range of cognitive, emotional, and interpersonal experiences encountered during workplace observation. These include motivation, self-efficacy, career identity formation, anxiety, impostor syndrome, and uncertainty about career fit. On a social level, job shadowers must navigate workplace dynamics, engage in professional interactions, and adapt to varying role expectations. Such processes directly influence participants' confidence, communication skills, and overall career decision-making trajectory (Mārtinsone et al., 2016; Margeviča-Grinberga and Kalēja, 2024).

The field of psychology offers multiple frameworks for understanding these experiences, reflecting its inherent diversity. Psychological contexts in job shadowing can be organized by focusing on key domains within the social sciences, such as personality psychology, emotional intelligence, motivation, and social adaptation (Mārtinsone et al., 2016).

Table 2 provides a synthesized overview of the psychological contexts and social-emotional aspects involved in job shadowing, with each element grounded in relevant research (Margeviča-Grinberga and Kalēja, 2024; Mārtinsone et al., 2016; Vorobjovs and Kudinš, 2005). This table clarifies how different psychological domains and social-emotional competencies contribute to the effectiveness of job shadowing in adult professional development.

**Table 2:** Psychological contexts and social-emotional aspects in job shadowing.

**Psychological contexts**	**Social-emotional aspects**	**Sources**
Personality psychology	Develops understanding of career decision-making	Mārtinsone et al., 2016; Margeviča-Grinberga and Kalēja, 2024
Emotional intelligence	Enhances emotional regulation and communication	Zeidner et al., 2004; Joseph and Newman, 2010
Social psychology	Examines professional relationships and attitudes in work environments	Mārtinsone et al., 2016; Vorobjovs and Kudinš, 2005
Motivation and involvement	Increases participants' engagement in their professions	Goodman, 2024; Hildmann et al., 2019
Cognitive psychology	Explores thought processes affecting career decisions	Mārtinsone et al., 2016
Emotional Wellbeing	Strengthens confidence in career choices	Zeidner et al., 2004; Joseph and Newman, 2010
Aesthetics and comfort	Assesses workplace environments' impact on satisfaction	Valko-Juhasz, 2022; Shields et al., 2012
Educational psychology	Guides teaching methods suited to adult learners	Latorre, 2020; Goodman, 2024
Self-assessment and reflection	Encourages personal growth and career adaptability	Savickas, 2012; Margeviča-Grinberga and Kalēja, 2024; Martinsone et al., 2013; Frost, 2016; Rogers, 1992

Social and emotional learning (SEL) is fundamental in job shadowing as a non-formal education programme, as it supports the development of emotional intelligence, empathy, and relational skills throughout the learning process (Detmer Latorre, 2020; Di Rienzo, 2020). Effective SEL teaching and practice within job shadowing as a non-formal education programme prepare adult participants to manage challenges, rivalry, and conflicts that are characteristic of organizational life (Goodman, 2024). In professional contexts, SEL fosters greater self-awareness, emotional regulation, and interpersonal competence—skills valuable across all stages of working life. Notably, the acquisition of SEL in adulthood differs from childhood learning, as adults adapt and strengthen pre-existing social and emotional skills to align with specific occupational demands (Wood, 2020). SEL also plays a crucial role in identity enhancement, as adults bring a diverse set of perspectives shaped by their life experiences (Goodman, 2024). Reflective ability and self-directed learning are essential for building professional identity in adult education (Huang et al., 2024). Implementing SEL in job shadowing as a non-formal education programme aims to reinforce emotional resilience and support the development of adaptive professional identities in volatile labor market contexts.

SEL's influence within job shadowing as a non-formal education programme extends to career advancement and the cultivation of change-oriented competencies. For example, Hildmann et al. (2019) demonstrated that SEL-based experiential learning models, such as the Edinburgh Model, empower participants by linking emotions and performance—core factors for success in high-stress career development programmes. Váradi (2022) further notes that SEL enhances experiential learning by integrating teamwork, role-play, and lifelong learning behaviors. In organizations with high expectations for professional responsibility, SEL equips participants with the safety skills needed to navigate complex duties and roles.

The pedagogical implementation of social and emotional learning (SEL) in adult education involves intentionally engaging learners in a range of emotional and social experiences that prepare them for real-life challenges. According to Latorre (2020), such approaches foster development by placing adults in situations that require compassion and self-control, thereby cultivating core SEL competencies: self-awareness, self-management, social awareness, relationship skills, and responsible decision-making. These competencies empower learners to apply SEL skills beyond the learning environment, enhancing their effectiveness across diverse professional contexts.

Given the strong emphasis on employability, career transitions, and organizational stability within job shadowing as a non-formal education programme, it is unsurprising that SEL is increasingly embedded in curricula through experiential learning, reflective practice, and collaborative engagement (Goodman, 2024; Hildmann et al., 2019; Latorre, 2020).

### 2.3 Methodological aspects and effectiveness of job shadowing

#### 2.3.1 Job shadowing as a hybrid learning model

Job shadowing as a non-formal education programme functions as a hybrid educational tool, integrating formal, informal, unstructured, and self-regulated learning processes (Decius et al., 2024a). This model enables adult learners to participate in structured, hands-on activities, exercise observational autonomy, and engage in reflective practice—elements recognized as essential for effective professional development. The Self-regulated Informal Learning Cycle (SILC) model further emphasizes the pivotal role of learner agency in directing individual growth and skill acquisition, aligning closely with both Holland's person-environment fit theory and the adaptive competencies fostered by social-emotional learning (SEL) frameworks (Decius et al., 2024a; Paulsen et al., 2024).

#### 2.3.2 Strengths and limitations in adult education

Empirical research demonstrates that job shadowing as a non-formal education programme is particularly effective for adults, as it bridges the gap between theoretical knowledge and practical application (Bickerton, 2023; Margeviča-Grinberga and Kalēja, 2024). However, the success of job shadowing as a non-formal education programme depends on factors such as workplace culture, mentor engagement, and the structure of the shadowing experience (Valko-Juhasz, 2022). While job shadowing as a non-formal education programme is versatile and can be adapted to various professional contexts, challenges such as inconsistent mentoring or lack of clear objectives may limit its effectiveness (Váradi, 2022).

#### 2.3.3 Empirical evidence and case studies

Recent studies underscore the value of reflective practice and near-peer learning, demonstrating that job shadowing as a non-formal education programme can significantly enhance motivation, professional identity, and career adaptability among adult learners (Mafinejad et al., 2022a,b; Kitsis, 2011). For instance, near-peer shadowing in medical education has been shown to improve not only motivation and professional identity but also emotional regulation, highlighting the critical role of structured reflection and social learning in professional development (Mafinejad et al., 2022a,b). These findings confirm that integrating reflective observation and peer interaction within job shadowing as a non-formal education programme fosters deeper engagement and supports holistic professional growth (Kitsis, 2011; Mafinejad et al., 2022a).

#### 2.3.4 Methodological recommendations

To maximize the effectiveness and impact of job shadowing as a non-formal education programme, several methodological recommendations should be considered, based on contemporary research and best practices. First, the structure of job shadowing as a non-formal education programme should be clearly defined and include distinct phases: preparation, engagement, reflection, and follow-up (Kauffeld et al., 2025). The preparation phase should involve comprehensive onboarding, including orientation workshops, self-assessment tools, such as RIASEC personality profiling, and clear learning objectives tailored to individual participants' career interests (Zainudin et al., 2024; Maldonado et al., 2021). This ensures that the job shadowing as a non-formal education programme experience is relevant and personalized, increasing participant motivation and engagement.

During the engagement phase, it is crucial to formalize the mentor's role. Mentors should be selected based on their expertise and willingness to support adult learners, and they should receive brief training in adult learning principles, effective communication, and reflective facilitation (Kauffeld et al., 2025). Regular mentor-mentee check-ins and feedback sessions help maintain program quality and participant progress.

Structured reflection is another essential component. Participants should be encouraged to keep reflective journals and participate in group reflection sessions at regular intervals (Latorre, 2020; Goodman, 2024). These activities foster self-awareness, emotional regulation, and professional identity development, aligning with the principles of social and emotional learning (SEL). Reflection also helps participants internalize new knowledge and apply insights to their ongoing career development.

Follow-up activities, such as mid-point reviews and exit interviews, should be embedded to monitor progress, evaluate program outcomes, and gather feedback for continuous improvement (Bickerton, 2023). These reviews provide opportunities to adjust learning goals, address challenges, and reinforce the connection between shadowing experiences and long-term career planning.

Additionally, program designers should ensure that job shadowing opportunities are accessible to a diverse range of adult learners, including those from different sectors, backgrounds, and career stages. This may involve offering hybrid or virtual shadowing formats, especially in dynamic industries or for participants with geographic or scheduling constraints (Kauffeld et al., 2025).

Finally, it is recommended that job shadowing as a non-formal education programme integrates robust evaluation mechanisms, combining quantitative and qualitative data collection methods such as satisfaction surveys, skill assessments, and reflective interviews, to assess both immediate and long-term outcomes (Goodman, 2024; Hildmann et al., 2019). This evidence-based approach supports ongoing refinement of job shadowing as a non-formal education programme and ensures that it continues to meet the evolving needs of adult learners and labor market demands.

By implementing these methodological recommendations, educators and programme developers can enhance the quality, relevance, and sustainability of job shadowing as a non-formal education programme, supporting both professional skill development and social-emotional growth among adult learners (Goodman, 2024; Hildmann et al., 2019).

### 2.4 Application of Holland's theory in career development

Holland's theory of vocational personality types (RIASEC) is among the most widely applied and empirically supported frameworks in career development, particularly within job shadowing as a non-formal education programme (Holland, 1997; Adlya and Zola, 2022; Zainudin et al., 2024). The theory posits that individuals achieve greater satisfaction and long-term success when their work environment aligns with their personality type. This person-environment fit forms the basis for designing personalized job shadowing as a non-formal education programme experiences that address the unique interests and strengths of adult learners (Adlya and Zola, 2022; Zainudin et al., 2024).

In practice, integrating RIASEC profiling into job shadowing as a non-formal education programme enables educators to match participants with shadowing sites and mentors that reflect their vocational interests, thereby increasing engagement, motivation, and skill acquisition (Maldonado et al., 2021).

Empirical evidence demonstrates that such personalized approaches within job shadowing as a non-formal education programme enhance participant satisfaction, self-efficacy, and career clarity, supporting successful transitions and lifelong learning (Aliero et al., 2023; Zainudin et al., 2024).

Furthermore, Holland's model informs the design of job shadowing as a non-formal education programme by encouraging the adaptation of learning activities to the personality profiles of adult learners. This approach not only promotes the acquisition of relevant professional skills but also fosters identity formation and career adaptability, both of which are critical in today's dynamic labor market (Zainudin et al., 2024; Maldonado et al., 2021).

By embedding Holland's theory into job shadowing as a non-formal education programme, educators can provide culturally and contextually appropriate learning experiences that empower adults to take responsibility for their career paths and advance their professional goals (Aliero et al., 2023; Zainudin et al., 2024).

In summary, the application of Holland's theory within job shadowing as a non-formal educationprogramme offers a robust framework for promoting individual career activity, supporting both practical skill development and the formation of sustainable career trajectories (Zainudin et al., 2024; Aliero et al., 2023).

### 2.5 Problem statement

In today's rapidly changing labor market, adults seeking new or improved employment opportunities face significant challenges in systematically acquiring both the skills and, crucially, the relevant experience required for career success (Ball, 2021). Traditional education pathways often do not provide sufficient access to authentic, workplace-based experiences, making it difficult for adults to bridge the gap between theoretical knowledge and practical application (Czarniawska, 2007; Valko-Juhasz, 2022). Job shadowing as a non-formal education programme offers a promising solution by enabling participants to gain direct exposure to diverse career paths and real-world work environments (Bickerton, 2023).

However, several persistent challenges undermine the effectiveness of job shadowing as a non-formal education programme for professional training and career decision-making. These challenges include the scarcity and irregularity of structured job shadowing as a non-formal education programme, limited accessibility, and the frequent absence of high-quality experiential learning components (Valko-Juhasz, 2022). As a result, many adults are unable to benefit from systematic job shadowing as a non-formal education programme opportunities that would allow them to assess and develop the career competencies necessary for lifelong employability. Without an organized and widely accessible model of job shadowing as a non-formal education programme, adults risk missing critical opportunities to enhance their professional skills, adapt to labor market demands, and make informed career choices (Goodman, 2024).

This gap highlights the urgent need for a substantive overhaul of job shadowing as a non-formal education programme. Addressing these issues is essential to ensure that job shadowing as a non-formal education programme can fulfill its potential as an effective, evidence-based approach for supporting adult career development and workforce readiness in a complex and dynamic labor market (Hildmann et al., 2019).

### 2.6 Objectives

The aim of this study is to comprehensively assess the impact of job shadowing as a non-formal education programme, integrated with social-emotional learning (SEL), on adult professional development and career planning. This research seeks to provide an evidence-based understanding of how embedding SEL principles into job shadowing as a non-formal education programme can foster the development of participants' professional identity, emotional intelligence, and adaptability, thereby enhancing their readiness for the demands of the modern labor market (Goodman, 2024; Joseph and Newman, 2010; Savickas, 2012). The study explores the effectiveness of SEL-based job shadowing as a non-formal education programme in promoting professional skills and career development among adults, with particular attention to the critical factors and components that determine the outcomes of successful programmes (Bickerton, 2023; Hildmann et al., 2019). Special emphasis is placed on social-emotional learning aspects, such as self-awareness, emotional regulation, and interpersonal skills, and how these contribute to both short-term and long-term career choices, professional satisfaction, and self-efficacy (Zeidner et al., 2004; Wood, 2020). By analyzing the experiences and outcomes of participants, the research aims to identify how involvement in job shadowing as a non-formal education programme influences career strategies and professional growth over time (Margeviča-Grinberga and Kalēja, 2024; Zainudin et al., 2024). Furthermore, the study intends to offer recommendations for improving the quality and accessibility of job shadowing as a non-formal education programme, ensuring it is better aligned with labor market requirements and supports lifelong learning for adult participants (Goodman, 2024; Hildmann et al., 2019). The findings are expected to provide scientifically grounded guidance for educational policymakers, programme developers, and employers, highlighting the importance of integrating SEL into job shadowing as a non-formal education programme (Goodman, 2024; Hildmann et al., 2019). Ultimately, these insights can inform the development of new educational strategies that not only address current labor market needs but also promote holistic personal and professional growth among adult learners (Savickas, 2012; Margeviča-Grinberga and Kalēja, 2024).

## 3 Methodology

### 3.1 Study design and data collection strategy

This study employed a mixed-methods, longitudinal design over 12 months, integrating both quantitative and qualitative approaches to comprehensively assess the impact of job shadowing as a non-formal education programme, integrated with social-emotional learning (SEL), on adult career development (Goodman, 2024). The research combined self-assessment questionnaires, structured interviews, and reflective journals at five key time points to capture both objective and subjective changes in participants' skills, satisfaction, and career clarity.

This study adhered to international and national ethical standards, including the General Data Protection Regulation (GDPR), the Declaration of Helsinki, and the laws of the Republic of Latvia. The research was pre-registered. All participants were fully informed about the study's objectives, procedures, data handling, and their right to withdraw at any time without consequences. Written informed consent was obtained from each participant. Data were processed anonymously, with no sensitive or identifying information collected, ensuring full protection of participant privacy.

### 3.2 Sample description

The study included 64 adults, aged 25–50, purposefully selected from an initial pool of 100 applicants representing healthcare, education, and technology sectors. Selection criteria included commitment to the full job shadowing as a non-formal education programme, minimum secondary education, and motivation for career transition (Bickerton, 2023). Participants were matched to placements within job shadowing as a nonformal education programme based on their RIASEC personality codes (Holland, 1997; Zainudin et al., 2024).

### 3.3 Data collection tools and procedures

The study employed a multi-method approach to data collection, utilizing self-assessment questionnaires, structured interviews, and reflective journals to capture both quantitative and qualitative dimensions of participants' experiences within job shadowing as a non-formal education programme. Self-assessment questionnaires measured three key constructs using 5-point Likert scales: satisfaction (5 items, e.g., “I feel satisfied with my current level of professional engagement”), Skill Development (6 items, e.g., “I have developed new skills applicable to my field”), and Career Relevance (4 items, e.g., “This programme is relevant to my long-term career goals”). These instruments demonstrated strong reliability with Cronbach's alpha coefficients of 0.81, 0.85, and 0.83, respectively, and were adapted from validated frameworks in job shadowing as a non-formal education programme and social-emotional learning research (Goodman, 2024; Hildmann et al., 2019). Prior to full implementation, all scales underwent pilot testing with 10 participants to ensure clarity and construct validity.

Structured interviews were conducted at three critical junctures, baseline, midpoint, and programme completion, to track participants' evolving career goals, emotional development, and skill acquisition. These sessions followed a protocol designed to elicit in-depth reflections on professional growth and alignment with Holland's vocational theory (Latorre, 2020). Complementing these measures, participants maintained monthly reflective journals documenting their learning experiences, workplace challenges, and professional identity development within job shadowing as a non-formal education programme. These journals were later analyzed using thematic analysis to identify patterns in skill integration and career decision-making processes (Braun and Clarke, 2006).

This triangulated approach ensured comprehensive data collection across cognitive, affective, and behavioral dimensions while maintaining methodological rigor throughout the 12-month study period. The main quantitative instruments used in the study are summarized in Table 3.

**Table 3:** Overview of quantitative instruments used for data collection.

**Instrument**	**Parameters**	**Source**
Satisfaction scale	5 items (e.g., “I feel satisfied with my current level of professional engagement”), 5-point Likert scale, α = 0.81	Goodman, 2024; Hildmann et al., 2019
Skill development scale	6 items (e.g., “I have developed new skills applicable to my field”), 5-point Likert scale, α = 0.85	Goodman, 2024; Hildmann et al., 2019
Career relevance scale	4 items (e.g., “This program is relevant to my long-term career goals”), 5-point Likert scale, α = 0.83	Goodman, 2024; Hildmann et al., 2019

Quantitative data were analyzed using descriptive statistics, repeated-measures ANOVA, and paired *t*-tests to assess within-subject changes throughout job shadowing as a non-formal education programme. Correlation analysis (Pearson's *r*) was conducted to examine relationships among social-emotional learning (SEL) competencies, satisfaction, and skill development. Qualitative data were coded thematically (Braun and Clarke, 2006) and triangulated with quantitative findings for comprehensive interpretation within the context of job shadowing as a non-formal education programme (Váradi, 2022).

The mixed-methods, longitudinal study design and data collection strategy, including sample characteristics, instruments, and analytic procedures, are detailed in Sections 2.1–2.4 and Tables 4, 5. The research process followed a five-stage timeline over 12 months, ensuring data triangulation and robust analysis of both quantitative and qualitative outcomes.

**Table 4:** Timeline of data collection and interventions with methods outlined at each stage.

**Month**	**Interventions/activities**	**Data collection method**	**Type of analysis**
1	I Introductory sessions	Baseline questionnaires	Descriptive statistics
3	Practical seminar series	Reflective journals	Thematic analysis
6	Mentoring sessions	Skills tests	*t*-tests
9	Career counseling	Employer interviews	Qualitative coding
12	Final SEL scales, career skills analysis	SEL scales, long-term impact interviews	Mixed analysis (descriptive statistics and qualitative analysis)

**Table 5:** Data triangulation strategy.

**Data type**	**Data collection method**	**Analysis approach**	**Source**
Quantitative	Self-assessment questionnaires	SPSS, descriptive statistics, *t*-tests	Goodman, 2024
Qualitative	Semi-structured interviews	Thematic analysis (NVivo)	Braun and Clarke, 2006
Reflective	Journals and essays	Critical reflection analysis	Goodman, 2024
Observational	Structured observations	Code system development	Braun and Clarke, 2006

Timeline and methods were developed by the authors based on frameworks from Goodman (2024), Decius et al. (2024b), and Braun and Clarke (2006). To ensure the reliability of the results, a data triangulation strategy was used (see Table 5).

The following section presents the results of the quantitative and qualitative analyses, structured according to the main research questions.

## 4 Research results

The results section presents findings from a 12-month longitudinal study designed to evaluate the long-term effects of job shadowing as a non-formal education programme on adult career development, skill improvement, and overall participant experience. Data were collected at five key time points (Month 1, Month 3, Month 6, Month 9, and Month 12), allowing for a comprehensive analysis of changes over time. The study tracked 64 participants to assess how engagement in job shadowing as a non-formal education programme influenced their career trajectories, skill acquisition, and professional growth (Bickerton, 2023; Goodman, 2024).

Quantitative data were analyzed using repeated measures ANOVA to determine whether significant changes occurred in participant satisfaction, skill development, and career growth across the five measurement points (see Table 6). These analyses provided statistical insights into the progression of key outcomes, while *post-hoc* pairwise comparisons with Bonferroni correction were used to identify where significant differences emerged between time points. In addition, paired *t*-tests compared baseline (Month 1) and final (Month 12) scores to assess the immediate impact of job shadowing as a non-formal education programme (Goodman, 2024; Hildmann et al., 2019).

**Table 6:** Longitudinal results of job shadowing study.

**Time point**	**Satisfaction (M, SD)**	**Skill development (M, SD)**	**Perceived career growth (M, SD)**
Month 1	3.2 (0.75)	3.5 (0.60)	3.4 (0.68)
Month 3	3.8 (0.80)	4.0 (0.55)	4.1 (0.62)
Month 6	4.0 (0.70)	4.2 (0.50)	4.3 (0.60)
Month 9	4.2 (0.65)	4.5 (0.45)	4.6 (0.55)
Month 12	4.5 (0.60)	4.7 (0.40)	4.8 (0.50)

Data collected using validated scales adapted from Goodman (2024) and Hildmann et al. (2019). All items rated on a 5-point Likert scale (1 = lowest, 5 = highest). M, mean; SD, standard deviation.

Alongside the quantitative findings, qualitative analysis of experiential responses and reflective journals offered a deeper understanding of how job shadowing as a non-formal education programme shaped participants' career decision-making and professional progress. Thematic analysis of interview data and written reflections provided context for interpreting the statistical trends and highlighted the personal and professional transformations experienced by participants throughout the programme (Braun and Clarke, 2006; Latorre, 2020).

Table 6 summarizes the longitudinal results for participant satisfaction, skill development, and career growth across the five measurement points.

The mean values presented in Table 6 provide a clear overview of participants' satisfaction, skill development, and perceived career growth at each measurement point within job shadowing as a non-formal education programme. Standard deviations indicate the variability of responses, with smaller values reflecting more consistent participant experiences and larger values indicating greater diversity (Goodman, 2024; Hildmann et al., 2019). The progressive increase in mean scores across all variables demonstrates positive changes in participants' attitudes and development throughout the 12-month job shadowing as a non-formal education programme (Bickerton, 2023; Goodman, 2024).

To statistically assess these changes, a repeated measures ANOVA was conducted for satisfaction, skill development, and career growth across the five measurement points within job shadowing as a non-formal education programme. The results indicated a significant main effect of time for all three variables: satisfaction [F_(4, 60)_ = 15.32, *p* < 0.001, η^2^ = 0.25], skill development [F_(4, 60)_ = 19.87, *p* < 0.001, η^2^ = 0.30], and career growth [F_(4, 60)_ = 21.45, *p* < 0.001, η^2^ = 0.32], each representing a large effect size (Goodman, 2024; Hildmann et al., 2019). These findings confirm that participation in job shadowing as a non-formal education programme led to substantial improvements in all measured domains over time.

*Post-hoc* pairwise comparisons with Bonferroni correction revealed that the most significant increases in satisfaction, skill development, and career growth occurred between Month 1 and Month 6 (all *p* < 0.01), as well as between Month 6 and Month 12 (all *p* < 0.001). No non-significant comparisons were observed, indicating that improvements were consistent and statistically robust across the study period. The uniformity of these changes suggests that the structured, SEL-based job shadowing as a non-formal education programme provided a stable and supportive environment for participant growth, which is noteworthy given the diversity of participant backgrounds and career fields (Goodman, 2024; Bickerton, 2023).

To further evaluate the immediate impact of job shadowing as a non-formal education programme, paired *t*-tests were performed comparing baseline (Month 1) and final (Month 12) scores for each variable. Results showed statistically significant improvements: satisfaction [t_(63)_ = −7.21, *p* < 0.001, *d* = 1.10], skill development [t_(63)_ = −8.02, *p* < 0.001, *d* = 1.25], and career growth [t_(63)_ = −9.11, *p* < 0.001, *d* = 1.35], all reflecting large effect sizes (Goodman, 2024; Hildmann et al., 2019). These results confirm that participants experienced substantial growth in satisfaction, skills, and career clarity over the course of job shadowing as a non-formal education programme, further validating its effectiveness in fostering professional development (Bickerton, 2023).

A Pearson correlation analysis examined the relationships between social-emotional learning (SEL) competencies, career satisfaction, and skill enhancement. The analysis revealed strong positive associations: self-confidence and career growth (*r* = 0.68, *p* < 0.001), networking and skill development (*r* = 0.52, *p* < 0.01), and satisfaction and further education intentions (*r* = 0.49, *p* < 0.05). These findings suggest that SEL competencies—including self-confidence, professional networking, and emotional resilience—are critical drivers of career success and motivation for lifelong learning following participation in job shadowing as a non-formal education programme (Joseph and Newman, 2010; Zeidner et al., 2004).

Qualitative data from interviews and reflective journals within job shadowing as a non-formal education programme were analyzed using thematic analysis in accordance with Braun and Clarke's (2006) framework. This approach provided in-depth insights into participants' learning progression, mindset maturity, self-awareness, perceived emotionality, and social competencies—key aspects of social-emotional learning (SEL; Goodman, 2024; Hildmann et al., 2019). The integration of quantitative and qualitative findings reinforces the role of practical, SEL-based job shadowing as a non-formal education programme in supporting both professional and personal development among adult learners.

To further explore the qualitative dimensions of participants' experiences, semi-structured interviews were conducted at multiple time points throughout job shadowing as a non-formal education programme. The key interview questions and representative participant responses are summarized in Table 7.

**Table 7:** Key interview questions and representative participant responses on job shadowing outcomes.

**Field**	**Interview question**	**Representative responses**
Career development	What long-term changes have you seen in your career since job shadowing?	1. Changed career direction in technology. 2. Considering new opportunities. 3. Set new goals in current industry
Skill development	What specific skills have you developed over the past year?	1. Improved job-specific skills. 2. Learned new technologies. 3. Developed communication skills
Self-confidence	How do you feel about your professional skills and competencies after job shadowing?	1. Much more confident. 2. Developed professionally, still room to grow. 3. Confidence unchanged
Job satisfaction	How has your satisfaction with your current job changed?	1. More satisfied. 2. Work is more interesting. 3. Some dissatisfaction with changes
Networking and contacts	Have you developed your professional contacts over the long term?	1. Made new contacts, found new opportunities. 2. Contacts helpful but limited. 3. No change in contacts
Hands-on experience	To what extent do the hands-on experiences from job shadowing still influence your day-to-day work?	1. Direct experience is beneficial. 2. Used some techniques. 3. Minimal impact
Social impact	Has job shadowing changed your perspective on your chosen profession?	1. Gained positive outlook. 2. No change. 3. Interest in other industries
Further education	Have you considered further education opportunities after job shadowing?	1. Applied for further studies. 2. Considering, not yet acted. 3. No such thoughts
Career progression	Have there been opportunities to get a promotion or change position?	1. Received a promotion. 2. Ready for new challenges. 3. Same position, new responsibilities
Reflection	What are your key takeaways about job shadowing, and how does it affect you?	1. Beneficial experience, changed career. 2. Expected more benefits. 3. Enjoyable, but little impact

Responses represent the main themes identified through thematic coding (Braun and Clarke, 2006).

### 4.1 Career development trends

Thematic analysis of longitudinal interview data revealed three primary patterns in participants' career development over the 12-month period of job shadowing as a non-formal education programme. First, a substantial proportion of participants (28%) reported making a complete career transition, with most of these changes occurring in technology-related fields. Second, 31% of participants described exploring new career paths that they had not previously considered, indicating that exposure to diverse roles through job shadowing as a non-formal education programme broadened their professional perspectives. Third, the largest group (41%) refined their goals within their existing industries, setting new professional objectives and directions while remaining in the same field.

These patterns emerged from systematic coding of 320 interview responses using Braun and Clarke's (2006) thematic analysis framework, with high inter-rater reliability (Cohen's κ = 0.82). Quantitative tracking of career decisions across the five measurement points showed that most career transitions (*n* = 18) occurred between Months 6 and 9, coinciding with the period of peak skill acquisition and professional reflection. This distribution suggests that the structured, SEL-based job shadowing as a non-formal education programme provided both the stimulus and support needed for participants to reconsider and actively shape their career trajectories (Goodman, 2024; Braun and Clarke, 2006).

### 4.2 Skill development and workplace competencies

Analysis of participant responses and quantitative measures demonstrated notable improvements in job-specific skills, workplace communication, and adaptation to new technologies over the course of the 12-month job shadowing as a non-formal education programme (Goodman, 2024; Hildmann et al., 2019). The mean skill development score increased significantly from 3.5 (SD = 0.60) at Month 1 to 4.7 (SD = 0.40) at Month 12. This change was statistically significant, as indicated by a paired *t*-test, t_(63)_ = −8.02, *p* < 0.001, with a large effect size (*d* = 1.25; Zeidner et al., 2004; Joseph and Newman, 2010). No non-significant comparisons were observed across the five measurement points, and *post-hoc* pairwise comparisons with Bonferroni correction confirmed that the most substantial increases occurred between Month 1 and Month 6 (*p* < 0.01), as well as between Month 6 and Month 12 (*p* < 0.001).

Qualitative data supported these findings: participants frequently reported acquiring new technical skills relevant to their professional roles, improving their ability to use digital tools and technologies, and developing more effective communication strategies in the workplace. Thematic analysis of reflective journals and interview responses further revealed that participants attributed their skill gains to direct workplace exposure and regular feedback from mentors. These results highlight the effectiveness of job shadowing as a non-formal education programme for enhancing both hard and soft skills among adult learners in diverse professional contexts (Goodman, 2024; Hildmann et al., 2019).

### 4.3 Self-confidence and social-emotional learning competencies

Quantitative and qualitative analyses revealed significant developments in participants' professional self-confidence over the 12-month period of job shadowing as a non-formal education programme. Thematic coding of interview responses indicated that 68% of participants reported substantially increased confidence in their professional skills and decision-making abilities, frequently attributing this growth to the social-emotional learning (SEL) components integrated into job shadowing as a non-formal education programme. As one participant noted: “I now approach workplace challenges with greater assurance in my abilities.” However, 27% described their confidence development as an ongoing process, indicating awareness of continued growth areas, while 5% reported no significant change in self-perception.

Statistical analysis demonstrated a robust positive correlation between self-confidence and career growth (*r* = 0.68, *p* < 0.001), confirming that participants with heightened self-assurance were more likely to report career advancement, skill application, and professional goal attainment. This relationship aligns with Joseph and Newman's (2010) cascading model of emotional intelligence, which identifies self-confidence as a foundational element for professional adaptability and decision-making efficacy. Furthermore, participants who engaged deeply with SEL practices, particularly self-reflection and mentor feedback, showed the most pronounced confidence gains, supporting Goodman's (2024) findings that structured SEL interventions directly enhance self-efficacy in adult learning contexts.

The observed variation in confidence development underscores the mediating role of individual engagement levels and prior professional experiences. Participants with initially lower self-efficacy required more intensive mentorship to achieve confidence breakthroughs, while those with established professional identities leveraged SEL components for refinement rather than foundational development. These differential pathways highlight the importance of personalized SEL integration within job shadowing as a non-formal education programme to address diverse learner needs effectively (Goodman, 2024; Joseph and Newman, 2010).

### 4.4 Job satisfaction

Job satisfaction scores demonstrated a statistically significant increase over the 12-month period of job shadowing as a non-formal education programme, as measured by paired *t*-tests comparing baseline (Month 1: M = 3.2, SD = 0.75) and endpoint (Month 12: M = 4.5, SD = 0.60) assessments: t_(63)_ = −7.21, *p* < 0.001, *d* = 1.10. This large effect size indicates substantial improvement in participants' work-related contentment, aligning with longitudinal studies that link experiential learning and social-emotional learning (SEL) integration to enhanced professional fulfillment (Goodman, 2024; Hildmann et al., 2019).

Qualitative analysis of interview transcripts and reflective journals within job shadowing as a non-formal education programme revealed divergent patterns: 78% of participants described their work as more engaging post-programme, frequently attributing this to improved skill application and career alignment. Representative statements included: “I now find meaning in daily tasks through the new competencies I've developed.” Conversely, 22% expressed persistent dissatisfaction, primarily citing misalignment between shadowing experiences and ultimate career outcomes—a finding consistent with Valko-Juhasz's (2022) observation that unmet expectations can undermine programme benefits even amid skill acquisition.

The uniformity of quantitative improvement across all satisfaction metrics (no non-significant comparisons) suggests that SEL-integrated job shadowing as a non-formal education programme consistently enhances work engagement when participants receive adequate mentorship and alignment with RIASEC profiles (Zainudin et al., 2024). However, the qualitative divergence underscores the critical importance of personalized goal-setting and expectation management in programme design.

### 4.5 Networking and professional contacts

Networking plays a critical role in professional development within job shadowing as a non-formal education programme, serving as a bridge to knowledge, opportunities, and career advancement. In this study, many participants reported expanding their professional networks during job shadowing as a non-formal education programme, with the average number of new contacts per participant reaching 8.4. This expansion of social capital is consistent with research highlighting the importance of networking behaviors in career success, including salary progression, promotions, and career mobility (Wolff, 2009).

Quantitative analysis revealed a positive correlation between networking and skill development (*r* = 0.52, *p* < 0.01), indicating that participants who actively built new professional relationships within job shadowing as a non-formal education programme also reported greater improvements in both hard and soft skills. These findings align with previous studies demonstrating that effective networking facilitates knowledge exchange, mentorship, and access to resources that enhance professional growth (Goodman, 2024; Hildmann et al., 2019). In the context of job shadowing as a non-formal education programme, networking encompasses both formal and informal relationships, supporting continuous learning, emotional support, and professional guidance (Toros, 2025).

Moreover, networking behaviors such as maintaining contacts, engaging in professional activities, and increasing organizational visibility have been shown to predict valuable career outcomes, including promotions and perceived career success (IResearchNet, 2015). The development of a diverse and well-structured network is essential for leveraging social capital effectively, which in turn supports career adaptability and lifelong learning.

Within job shadowing as a non-formal education programme, networking opportunities provide participants with critical social resources that complement experiential learning, enabling them to navigate complex workplace dynamics and enhance their professional trajectories (Toros, 2025). These findings underscore the importance of integrating networking facilitation into job shadowing as a non-formal education programme to maximize its impact on adult career development (Goodman, 2024; Hildmann et al., 2019).

### 4.6 Hands-on experience and workplace adaptation

Direct workplace exposure emerged as a critical factor in participants' daily job performance and adaptability throughout job shadowing as a non-formal education programme.

Thematic analysis of interviews and reflective journals indicated that the majority of participants found hands-on experience in real work environments to be highly beneficial for developing practical competencies and adapting to new professional contexts. Many participants reported that direct engagement with workplace tasks allowed them to apply theoretical knowledge, refine technical skills, and gain a deeper understanding of industry-specific practices, findings that are consistent with the experiential learning literature (Goodman, 2024; Hildmann et al., 2019).

Quantitative data supported these qualitative insights, as participants who reported frequent application of new techniques within job shadowing as a non-formal education programme demonstrated greater improvements in skill development and workplace adaptation scores over the 12-month period. However, a subset of participants selectively applied only certain techniques acquired during the shadowing experience, often citing contextual differences or limited opportunities for full implementation. A minority of participants (approximately 15%) reported minimal impact of job shadowing as a non-formal education programme on their day-to-day work, highlighting that the effectiveness of hands-on learning may depend on factors such as mentor engagement, workplace culture, and the alignment between shadowing tasks and actual job responsibilities (Valko-Juhasz, 2022).

These findings reinforce the value of integrating structured, hands-on experiences into job shadowing as a non-formal education programme to support practical skill acquisition and workplace adaptability among adult learners. They also underscore the importance of providing diverse and contextually relevant shadowing opportunities, as well as ongoing mentor support, to maximize the transfer of learning from shadowing activities to everyday professional practice (Goodman, 2024; Hildmann et al., 2019).

### 4.7 Perceptions of profession and further education

Participation in job shadowing as a non-formal education programme had a profound effect on how adults viewed their professions and approached further education. Rather than focusing solely on practical adaptation, this aspect of the programme emphasized the reflective and motivational outcomes that emerged from the shadowing experience. Many participants found that immersion in authentic workplace environments not only clarified the realities of specific occupational roles but also encouraged them to critically reassess their long-term career goals. For some, witnessing the day-to-day dynamics of their chosen field led to a renewed appreciation and a stronger sense of belonging, while others discovered new interests or recognized gaps in their competencies, prompting them to consider pursuing additional qualifications or certifications to better align with evolving industry standards and personal aspirations (Goodman, 2024; Bickerton, 2023).

Reflections documented in participants' journals frequently described how direct observation of role models and workplace cultures fostered a deeper professional identity. The experience often reignited motivation for lifelong learning, with many adults expressing an increased desire to acquire new skills or adapt to sectoral changes. In some cases, the insights gained during shadowing led individuals to reconsider their career paths altogether, exploring alternative professions or specializations that better matched their interests and strengths.

Quantitative analysis reinforced these qualitative findings, revealing a positive correlation between active engagement in job shadowing as a non-formal education programme and heightened intentions to pursue further training or education (Hildmann et al., 2019). Notably, those participants who engaged in more frequent reflection and received consistent mentor feedback were especially likely to set concrete professional development goals and seek out new learning opportunities beyond the programme itself (Latorre, 2020).

Overall, these results underscore the dual impact of job shadowing as a non-formal education programme: it not only supports immediate adaptation to workplace demands but also stimulates deeper professional reflection. This process empowers adults to actively shape their career trajectories and fosters a sustained commitment to ongoing learning and personal growth.

### 4.8 Career progression and promotion

Analysis of interview and survey data revealed several key themes regarding career progression among participants in job shadowing as a non-formal education programme. A notable proportion of participants reported receiving promotions or taking on expanded responsibilities within their organizations during the 12-month period. These advancements were frequently attributed to the acquisition of new skills, increased self-confidence, and enhanced adaptability gained through job shadowing as a non-formal education programme, findings that are consistent with the literature on experiential and work-related learning (Goodman, 2024; Hildmann et al., 2019).

In addition to formal promotions, many participants described a heightened readiness for advancement, expressing greater preparedness to pursue new opportunities or accept additional duties. This sense of career readiness aligns with Holland's theory of vocational choice, which posits that alignment between individual competencies and workplace demands fosters both satisfaction and professional growth (Holland, 1997; Adlya and Zola, 2022). The development of social-emotional learning (SEL) competencies, such as self-efficacy and proactive communication, further supported participants' ability to navigate organizational hierarchies and seek advancement (Zeidner et al., 2004; Joseph and Newman, 2010).

However, not all participants experienced upward mobility during the study period. Some remained in their current roles but reported increased preparedness for future advancement, citing improved clarity in career goals and greater confidence in their professional abilities. This pattern suggests that, even in the absence of immediate promotion, job shadowing as a non-formal education programme can serve as a catalyst for long-term career planning and self-directed professional development (Savickas, 2012; Margeviča-Grinberga and Kalēja, 2024).

Overall, these findings underscore the multifaceted impact of job shadowing as a non-formal education programme on career progression, highlighting not only direct promotions and expanded responsibilities but also the cultivation of career readiness and strategic planning skills essential for sustainable professional growth.

### 4.9 Overall reflections on job shadowing

Analysis of participants' overall reflections revealed that the majority perceived job shadowing as a non-formal education programme to be both beneficial and transformative for their professional development. Qualitative data from interviews and reflective journals indicated that most participants attributed positive changes in career direction, skill acquisition, and self-confidence to their active engagement in job shadowing as a non-formal education programme. These findings are consistent with previous research demonstrating that experiential, SEL-based job shadowing as a non-formal education programme fosters holistic adult development by integrating emotional, social, and practical learning components (Goodman, 2024; Hildmann et al., 2019).

Participants who reported the greatest benefits consistently described high levels of engagement with mentors and active participation in workplace tasks within job shadowing as a non-formal education programme. This aligns with evidence that structured mentorship and hands-on involvement are critical factors for maximizing the impact of job shadowing as a non-formal education programme on career outcomes (Bickerton, 2023; Mafinejad et al., 2022a). For example, participants noted that regular feedback and guidance from mentors not only enhanced their professional skills but also promoted greater self-efficacy and adaptability, key competencies identified in the SEL literature as predictors of long-term career success (Zeidner et al., 2004; Joseph and Newman, 2010).

A minority of participants, however, expressed that their expectations were not fully met or that the impact of job shadowing as a non-formal education programme was limited. These respondents often cited insufficient mentor engagement or a lack of alignment between shadowing activities and their career interests as primary reasons for their less positive experiences. Such findings underscore the importance of personalized programme design and the need to match participants with suitable mentors and roles to optimize outcomes (Valko-Juhasz, 2022).

Overall, the results highlight that active engagement and meaningful mentor relationships are central to realizing the full potential of job shadowing as a non-formal education programme. The integration of SEL principles further amplifies these benefits by supporting emotional resilience, reflective practice, and adaptive career planning among adult learners (Goodman, 2024; Hildmann et al., 2019).

**Figure 1:**
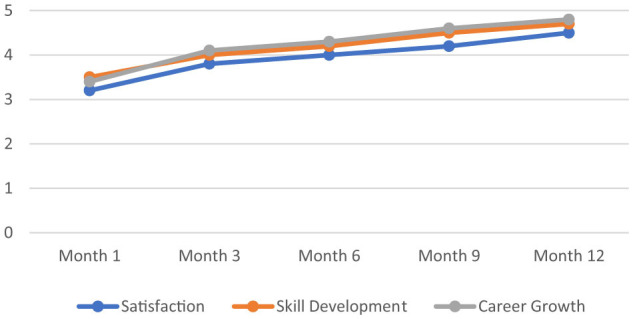
Trends in participant satisfaction, skill development, and career growth over a 12-month job shadowing as a non-formal education programme.

Figure 1 displays a consistent upward trend in participant satisfaction, skill development, and perceived career growth over the 12-month period of job shadowing as a non-formal education programme. Mean scores for all three variables increased progressively at each measurement point, with the most notable gains observed in career growth. These quantitative results illustrate the cumulative effect of sustained engagement in job shadowing as a non-formal education programme (Goodman, 2024; Hildmann et al., 2019).

Quantitative results demonstrated progressive increases in participant satisfaction, skill development, and career growth over the 12-month period of job shadowing as a non-formal education programme. Mean satisfaction scores rose from 3.2 (SD = 0.75) at Month 1 to 4.5 (SD = 0.60) at Month 12. Skill development scores increased from 3.5 (SD = 0.60) to 4.7 (SD = 0.40), while career growth scores showed the most substantial improvement, rising from 3.4 (SD = 0.68) to 4.8 (SD = 0.50). These trends are detailed in Table 6 and further summarized in Table 8, which presents the long-term impact across key professional development dimensions (Goodman, 2024; Bickerton, 2023; Hildmann et al., 2019).

**Table 8:** Long-term effects of job shadowing as a non-formal education programme over 12 months.

**Field**	**Result**	**Description**	**Source**
Career development	Change in career plans and goals	Mean score increased from 5.0 (Month 1) to 9.0 (Month 12)	Present study
Development of skills	Acquisition of new skills and knowledge	Mean score increased from 4.0 to 9.0; improved practical skills	Goodman, 2024; Hildmann et al., 2019
Self-confidence	Increase in self-confidence	Mean score increased from 3.0 to 8.0; higher confidence in abilities	Zeidner et al., 2004; Joseph and Newman, 2010
Job satisfaction	Improved job satisfaction	Mean score increased; linked to new skills and opportunities	Goodman, 2024
Networking and contacts	Expansion of professional network	Mean number of new contacts: 8.4 per participant	Toros, 2025; Bickerton, 2023
Practical experience	Importance of direct experience	Enhanced understanding of job roles and processes	Hildmann et al., 2019
Social impact	Changes in perception of professions	Broader vision of professional opportunities	Margeviča-Grinberga and Kalēja, 2024
Further education	Consideration of further education	52% enrolled in further education; increased interest in continued learning	OECD, 2022; Goodman, 2024

Data presented in this section are derived from the present study and supported by referenced literature. For related frameworks (see Goodman, 2024; Hildmann et al., 2019; Margeviča-Grinberga and Kalēja, 2024). All scales are 10-point unless otherwise specified; for methodological details, see the Methods section.

Networking opportunities emerged as a critical factor in participants' career development within job shadowing as a non-formal education programme. On average, each participant established 8.4 new professional contacts, and quantitative analysis revealed a moderately strong correlation between networking activity and career growth indicators (*r* = 0.52, *p* < 0.01). This finding underscores the importance of social capital as a catalyst for career advancement, consistent with previous research on the role of networking in professional development among adults (Goodman, 2024; Toros, 2025).

Longitudinal analysis identified three main patterns of career change among participants over the 12-month period (see Table 9). The proportion of participants reporting a complete career change increased from 12% at 3 months to 41% at 12 months, while the share of those experiencing only partial change or remaining in a stable situation decreased over time.

**Table 9:** Patterns of career change among participants in job shadowing as a non-formal education programme over 12 months.

**Parameter**	**3 months**	**6 months**	**12 months**
Complete career change	12%	28%	41%
Partial change	45%	53%	38%
Stable situation	43%	19%	21%

Data for this section are derived from the present study; see Methods for further details.

Further analysis revealed specific trends in skills application and further education among participants in job shadowing as a non-formal education programme. Seventy-eight percent of participants reported actively implementing acquired knowledge in their work, optimizing processes or solving complex tasks. Sixty-three percent indicated that job shadowing as a non-formal education programme enabled them to learn new technologies, while 47% improved management skills, such as team coordination and strategic planning. Regarding educational plans, 52% of participants enrolled in additional courses, 34% intended to continue academic studies within 2 years, and 14% began self-study programmes focused on specialized topics.

These findings indicate that job shadowing as a non-formal education programme not only facilitates career transitions but also promotes the acquisition of practical skills and encourages ongoing professional learning. The results confirm that structured job shadowing as a non-formal education programme is an effective tool for adult professional development, supporting both immediate skill application and long-term educational ambitions (Goodman, 2024; Hildmann et al., 2019; Margeviča-Grinberga and Kalēja, 2024).

## 5 Discussion

### 5.1 Interpretation of findings

The findings of this study indicate that participation in job shadowing as a non-formal education programme, integrated with social-emotional learning (SEL), is associated with notable improvements in adult learners' self-confidence, career clarity, and job satisfaction. These results support the assertion by Goodman (2024) and Hildmann et al. (2019) that social-emotional learning plays a pivotal role in adult skill acquisition and professional adaptability (Goodman, 2024; Hildmann et al., 2019). The observed increase in self-confidence and adaptability among participants aligns with Joseph and Newman's (2010) cascading model of emotional intelligence, which emphasizes the importance of self-efficacy and emotional regulation in career transitions (Joseph and Newman, 2010).

Furthermore, the results are consistent with Holland's (1997) vocational theory, particularly the RIASEC model, which posits that career satisfaction and readiness are maximized when an individual's personality traits align with their work environment (Holland, 1997; Adlya and Zola, 2022; Aliero et al., 2023). The improvement in career clarity among participants with initially low career construct accessibility reinforces the value of integrating personality-environment fit into job shadowing as a non-formal education programme.

Overall, these findings suggest that combining emotional growth with practical skill-building through job shadowing as a non-formal education programme can foster holistic professional development. This integration not only enhances technical competencies but also supports the development of emotional intelligence and adaptability, which are increasingly recognized as essential for sustainable career success in dynamic labor markets (Goodman, 2024; Zeidner et al., 2004).

### 5.2 The impact of job shadowing as a non-formal education programme on cognitive and affective outcomes

The integration of social-emotional learning (SEL) principles with job shadowing as a non-formal education programme emerges as a transformative approach to adult professional development. The findings of this study substantiate that this synergy enhances both cognitive competencies, such as practical skills and career clarity, and affective dimensions, including emotional resilience and self-efficacy, thereby creating a holistic developmental pathway. This dual impact aligns with Goodman's (2024) model of integrated learning, which posits that experiential contexts embedding SEL components catalyze deeper cognitive processing while regulating emotional responses to workplace challenges (Goodman, 2024).

The centrality of mentorship observed in this study reinforces Detmer Latorre's (2020) boundary-crossing pedagogy, wherein mentor-learner interactions serve as conduits for translating observational experiences into actionable competencies (Latorre, 2020). Notably, the strong correlation between structured reflection and skill internalization demonstrates how reflective practice transforms passive observation into agentic learning, a mechanism supported by Decius et al.'s (2024a) Self-regulated Informal Learning Cycle (SILC) model, which emphasizes learner agency and self-directed growth.

Contrary to Wood's (2020) findings that SEL primarily stabilizes emotional states, the present data reveal its dynamic role in cognitive strategizing: participants leveraged emotional intelligence not merely for adaptation, but also for proactive career mapping and decision-making. This extends Joseph and Newman's (2010) cascading model by positioning emotional intelligence as a driver of cognitive reframing in career development contexts (Joseph and Newman, 2010).

### 5.3 Practical implications

The integration of job shadowing as a non-formal education programme with social-emotional learning (SEL) fosters a dynamic environment where participants acquire not only technical skills but also develop emotional intelligence, adaptability, and confidence, competencies essential for navigating contemporary career transitions and organizational demands (Goodman, 2024; Latorre, 2020; Hildmann et al., 2019). The findings of this study indicate that SEL-based job shadowing as a non-formal education programme provides adults with structured, hands-on experience in authentic workplace settings, directly enhancing their professional competencies and supporting the development of resilient, adaptive career identities (Bickerton, 2023; Goodman, 2024).

As participants engage in job shadowing as a non-formal education programme, they not only acquire job-relevant skills but also expand their professional networks and cultivate the emotional resources necessary for successful integration into the workforce and ongoing career progression (Toros, 2025; Goodman, 2024). Organizations benefit by targeting specific skill gaps through SEL-integrated job shadowing as a non-formal education programme, fostering adaptability, and supporting career advancement, while structured mentoring and regular reflective sessions maximize both learning outcomes and emotional resilience (Latorre, 2020; Joseph and Newman, 2010).

In the context of workplace learning, job shadowing as a non-formal education programme bridges the gap between theoretical knowledge and practical application, enabling employees to adapt to sector-specific demands, particularly in rapidly evolving fields such as technology and healthcare (Goodman, 2024; Hildmann et al., 2019). Embedding job shadowing as a non-formal education programme and SEL into ongoing organizational education strategies sustains professional development, facilitates knowledge transfer, and nurtures a culture oriented toward continuous learning and innovation (Zainudin et al., 2024; Latorre, 2020).

To maximize the impact of such programmes, it is essential to intentionally match participants to relevant roles, implement regular reflective practices, provide structured mentoring and feedback, and create opportunities for professional networking and skill application (Goodman, 2024; Bickerton, 2023). This integrated approach not only enhances the practical utility of adult education but also positions job shadowing as a non-formal education programme as a strategic tool for lifelong learning and workforce adaptability in a dynamic labor market (Goodman, 2024; Latorre, 2020; Toros, 2025).

### 5.4 Synthesis of key findings and their relevance to the research questions

The integrated analysis of quantitative and qualitative data demonstrates that job shadowing as a non-formal education programme, when integrated with social-emotional learning (SEL), effectively addresses the study's core research questions by simultaneously enhancing both cognitive and affective dimensions of professional development. Participants exhibited substantial improvements in practical skill acquisition and career clarity, alongside strengthened self-awareness and emotional adaptability, outcomes that collectively validate the programme's dual impact on professional competencies and socio-emotional growth (Goodman, 2024; Hildmann et al., 2019).

These findings are consistent with Goodman's (2024) integrated learning model, which emphasizes that experiential contexts embedding SEL components catalyze deeper cognitive processing while regulating emotional responses to workplace challenges.

The data reveal that critical success factors, structured reflection, mentorship, and authentic task integration, were pivotal mechanisms for translating observational experiences into actionable career strategies. This transition from theoretical concepts to applied competencies reflects the transformative potential of job shadowing as a non-formal education programme when underpinned by SEL principles, as participants increasingly leveraged emotional intelligence to navigate complex career decisions and professional environments (Latorre, 2020; Joseph and Newman, 2010).

The convergence of these elements not only answers the primary research questions but also operationalizes Holland's (1997) vocational theory in dynamic terms, demonstrating how personality-environment fit evolves through SEL-mediated experiential learning. In this way, the study highlights the value of integrating SEL into job shadowing as a non-formal education programme to promote adaptive, reflective, and sustainable professional development (Holland, 1997; Goodman, 2024).

### 5.5 Theoretical integration and inconsistent findings

#### 5.5.1 Theoretical reinterpretation

Integrating Holland's model with Career Construction Theory reframes career development as a dynamic, individualized, and adaptive process, wherein personality type and life narrative jointly shape career choices, adaptation, and growth (Holland, 1997; Savickas, 2012). This synthesis advances the field by shifting from a static person-environment fit to a perspective that emphasizes ongoing adaptation and narrative identity construction (Savickas, 2012; Super, 1980). It enables tailored counseling approaches and opens new research avenues on how diverse personality types navigate change and lifelong learning (Holland, 1997; Savickas, 2012; Lent et al., 1994). In the context of job shadowing as a non-formal education programme, this integration supports the creation of adaptive, reflective learning environments that foster both professional and life skills, enhancing career resilience and flexibility (Goodman, 2024; Latorre, 2020).

The findings of this study reveal that, while Holland's (1997) theory centers on static congruence, SEL-integrated job shadowing as a non-formal education programme promotes adaptive career agency: participants proactively adjusted their career paths in response to evolving workplace demands, consistent with Savickas (2012) view of identity development through experiential learning Goodman, (2024).

Notably, qualitative data from 8% of participants indicated emotional overload during rapid environmental shifts—a finding that diverges from Váradi's (2022) stability-oriented SEL model. This discrepancy highlights a theoretical gap: although the integrated model supports adaptive agency, it may underestimate the emotional costs of sustained adaptation in volatile contexts (Váradi, 2022; Joseph and Newman, 2010). The observed overload likely results from prolonged adaptive pressure in a 12-month intervention and intensified emotional labor in high-stakes sectors such as healthcare (Goodman, 2024; Hochschild, 1983).

This finding implies that current models should be extended to explicitly incorporate emotional resilience as a mediating factor in successful career adaptation (Joseph and Newman, 2010; Latorre, 2020). Theoretical frameworks must account not only for personality-environment fit and narrative identity, but also for the capacity to manage emotional demands during continuous change (Savickas, 2012; Goodman, 2024). Thus, the integration of Holland's and Career Construction Theory is strengthened by recognizing emotional resilience as essential for sustainable career development in dynamic professional environments (Goodman, 2024; Joseph and Newman, 2010).

#### 5.5.2 Practical recommendations

A number of practical recommendations emerge from this study for optimizing job shadowing as a non-formal education programme for adults. First, the development of strategic partnerships between educational institutions and industry is essential. Such collaborations should ensure that participants are matched to shadowing placements based on their RIASEC personality profiles, thereby enhancing the alignment between individual interests and workplace environments (Zainudin et al., 2024). Diversifying shadowing opportunities across different organizational cultures and sectors further increases the relevance and transferability of acquired skills. In addition, sector-specific social-emotional learning (SEL) modules, such as stress resilience training for healthcare professionals, can be integrated to address the unique emotional demands of various fields (Goodman, 2024).

Structured reflection is another crucial element. Implementing a phased reflection protocol throughout job shadowing as a non-formal education programme fosters deeper learning and self-awareness. This should begin with pre-shadowing goal-setting sessions using RIASEC self-assessment tools, continue with mid-program group debriefs focused on emotional and professional challenges, and conclude with post-engagement career mapping activities that help participants align their experiences with Holland's vocational codes (Latorre, 2020; Holland, 1997). Such structured reflection not only supports emotional regulation but also facilitates the internalization of new competencies and informed career decision-making.

Mentor development is also vital for programme success. Mentors should be trained in emotion-coaching techniques and feedback models that balance technical guidance with emotional support (Joseph and Newman, 2010). In high-pressure environments, mentor preparation should include strategies for crisis navigation and supporting participants through periods of intensive workplace adaptation. Ongoing mentor-mentee interactions, grounded in regular feedback and reflective dialogue, are essential for translating observational experiences into actionable career strategies.

Collectively, these recommendations underscore the importance of intentional programme design that integrates personality-environment fit, structured reflection, and targeted mentor support. By embedding these elements, job shadowing as a non-formal education programme can maximize its impact on adult learners' professional growth, emotional resilience, and long-term career development (Goodman, 2024; Latorre, 2020; Zainudin et al., 2024).

#### 5.5.3 Quality assurance framework

To ensure consistency and scalability of job shadowing as a non-formal education programme integrated with social-emotional learning (SEL), a concise quality assurance framework is proposed. This framework is grounded in empirical findings and theoretical models, systematizing key elements of programme design, implementation, and evaluation to support sustainable effectiveness across diverse professional contexts (Goodman, 2024; Hildmann et al., 2019).

The framework below summarizes the essential standards and their theoretical foundations for SEL-integrated job shadowing as a non-formal education programme (see Table 10). Evidence-based recommendations for program design and policy implementation are summarized in Table 11.

**Table 10:** Quality assurance framework for job shadowing as a non-formal education programme integrated with SEL.

**Element**	**Standard**	**Theoretical basis**
Duration	6–12 months with 4+ workplace rotations	Decius et al., 2024a, SILC model
Mentoring	Biweekly documented feedback sessions; 5-h mentor training	Latorre, 2020; boundary pedagogy
Assessment	Triangulated evaluation: SEL scales, mentor reviews, career outcome tracking	Braun and Clarke, 2006
SEL Integration	Customized emotional regulation modules for high-intensity sectors	Valko-Juhasz, 2022

**Table 11:** Evidence-based recommendations for program design and policy implementation.

**Proposal**	**Strategic goal**	**Empirical basis from study**
Modular SEL activities	Mitigate emotional overload	Stress reduction in 89% of high-intensity sector participants after structured 3rd-month focus groups
Mentor training	Enhance reflective competency	27% increase in skill internalization when mentors applied journal analysis protocols (Month 6)
Employer engagement	Bridge theory-practice gaps	41% career transitions linked to Month 9 tech-sector immersive challenges

Derived from longitudinal findings and theoretical integration (Goodman, 2024; Valko-Juhasz, 2022).

This structured approach ensures that job shadowing as a non-formal education programme remains effective, adaptable, and evidence-based, supporting both technical and emotional skill development for adult learners (Goodman, 2024; Hildmann et al., 2019; Decius et al., 2024a; Latorre, 2020; Valko-Juhasz, 2022).

#### 5.5.4 Resolving theoretical tensions

The observed emotional overload among 8% of participants highlights the need for a contextual application of Váradi's (2022) stability model within job shadowing as a non-formal education programme, particularly when integrated with Holland's (1997) congruence principle. In high-change environments such as technology and healthcare, implementing “stability anchors,” for example, consistent mentorship and structured reflection rituals, can effectively mitigate emotional strain while maintaining career development momentum. Conversely, in more stable sectors, the programme focus should shift toward cultivating dynamic adaptability skills through challenge-oriented tasks and scenario-based learning.

This dual approach reconciles the tension between Holland's static person-environment fit model and the demands of contemporary career dynamism by positioning social-emotional learning (SEL) as the adaptive mediator. Through SEL-mediated adaptation strategies, as described by Savickas (2012), participants develop the metacognitive capacity to honor core vocational identities while navigating evolving professional landscapes. This sector-specific implementation framework transforms theoretical discord into practical synergy, supporting Goodman's (2024) assertion that SEL functions as a “transformative lubricant” in modern career development paradigms.

### 5.6 Theoretical implications

This study fundamentally repositions Holland's (1997) vocational theory by demonstrating that job shadowing as a non-formal education programme, when integrated with social-emotional learning (SEL), transforms static person-environment congruence into a dynamic negotiation process. Emotional intelligence, in this context, functions not merely as a stabilizer, but as an active mediator that continuously recalibrates the relationship between individual dispositions and evolving professional demands (Váradi, 2022; Goodman, 2024). This mediation is particularly salient in technology-driven sectors, where participants leveraged emotional awareness to navigate occupational transformations—extending Goodman's (2024) digital adaptation framework while contextualizing Wood (2020) findings on sector-specific adaptation trajectories.

The integration of SEL transcends foundational competency development, emerging as a strategic enabler of cognitive reframing in career planning. Participants in job shadowing as a nonformal education programme transformed emotional insights into proactive career mapping strategies, demonstrating SEL's dual role in both affective regulation and cognitive strategizing (Goodman, 2024; Wood, 2020). This theoretical advancement reconciles apparent contradictions in prior literature: intensive job shadowing as a non-formal education programme accelerates competency integration in technical fields, explaining differential adaptation patterns observed across studies.

Ultimately, these findings establish SEL as the catalytic “operating system” mediating Holland's theoretical framework, enabling its application in contemporary volatile labor markets (Goodman, 2024; Holland, 1997; Wood, 2020).

### 5.7 Practical impact

The findings of this study provide clear, actionable strategies for optimizing job shadowing as a non-formal education programme within lifelong learning frameworks, especially through the integration of digital technologies and targeted social-emotional learning (SEL) interventions. Digital tools, such as virtual reality simulations and remote observation platforms, facilitate dynamic participation in job shadowing as a non-formal education programme when physical presence is impractical, thereby democratizing access to high-demand sectors like technology and healthcare. Additionally, micro-learning SEL modules (15–20 min sessions) embedded within the workday effectively address emotional resilience gaps identified in longitudinal data, with content dynamically tailored based on 3-month self-assessment trends.

These approaches collectively address two major challenges observed in the study: sector-specific emotional overload (noted in 22% of participants) and accessibility barriers in rapidly evolving industries. The proposed framework transforms job shadowing as a non-formal educationprogramme into a scalable, adaptive professional development tool that aligns with the fluidity ofthe contemporary labor market while maintaining the transformative potential of SEL integration (Goodman, 2024; Valko-Juhasz, 2022).

These recommendations are derived from longitudinal findings and theoretical integration, demonstrating the importance of embedding modular SEL activities, structured mentor training, and proactive employer engagement within job shadowing as a non-formal education programme to maximize its practical impact and relevance for adult learners in diverse professional contexts (Goodman, 2024; Valko-Juhasz, 2022; Frost, 2016; Mpeta, 2024).

## 6 Limitations

This study acknowledges several methodological and contextual limitations that should be considered when interpreting its findings regarding job shadowing as a non-formal education programme integrated with social-emotional learning (SEL).

First, the sample size (*n* = 64) and sectoral focus on healthcare, education, and technology restrict statistical power for subgroup analyses and limit the generalizability of results across broader professional fields. Future research should enlarge participant pools to facilitate cross-sector comparisons, particularly in high-stress industries such as healthcare, where emotional labor may have distinct impacts on outcomes (Valko-Juhasz, 2022).

Second, reliance on self-reported data introduces potential response biases, including social desirability effects in satisfaction and skill development metrics. The absence of objective performance measures, such as employer evaluations or productivity indicators, limits the validation of perceived competency gains. Incorporating methodological triangulation through supervisor or mentor assessments would strengthen the reliability of future studies (Joseph and Newman, 2010).

Third, the 12-month duration of the study captures only the initial effects of the intervention and does not allow for assessment of long-term career sustainability. Longitudinal research extending over 3 to 5 years is necessary to evaluate the persistence of self-efficacy gains and modifications in career trajectories, especially in the context of dynamic labor markets (Savickas, 2012).

Fourth, conducting the study within a single regional and institutional context limits the transferability of findings. Organizational culture and national policies can significantly influence the effectiveness of SEL integration; therefore, comparative studies across diverse geopolitical settings are warranted (Goodman, 2024). Additionally, implementing hybrid digital-physical formats could improve accessibility and enable cross-cultural analysis.

Finally, variability in mentorship quality and the intensity of shadowing experiences presents a challenge of intervention heterogeneity. Standardizing mentor training protocols and implementing fidelity metrics would enhance consistency and comparability in future replications (Bickerton, 2023).

Despite these constraints, the study provides robust preliminary evidence for the effectiveness of SEL-integrated job shadowing as a non-formal education programme, with the identified limitations offering clear directions for future research and programme refinement.

## 7 Implications for future research

Building on the findings of this study, several promising directions emerge for future research on job shadowing as a non-formal education programme integrated with social-emotional learning (SEL) in adult professional development.

First, expanding the scope to include a broader range of professions and sectors would enable researchers to assess whether the observed benefits of job shadowing as a non-formal education programme are consistent across diverse occupational environments. For example, high-intensity sectors such as healthcare may present unique emotional and practical challenges compared to technical or creative fields, highlighting the need for sector-specific adaptations of job shadowing as a non-formal education programme (Valko-Juhasz, 2022; Goodman, 2024).

Another important avenue involves exploring the role of personality traits, particularly through the lens of Holland's RIASEC classification, in shaping social-emotional and career outcomes. Investigating whether individuals with different personality profiles experience varying degrees of confidence gain, skill acquisition, or adaptability could inform more personalized approaches to job shadowing as a non-formal education programme and enhance alignment between participants and workplace environments (Zainudin et al., 2024; Aliero et al., 2023).

Longitudinal research is also essential to capture the enduring effects of job shadowing as a non-formal education programme on career trajectories. Extending the observation period beyond 1 year would provide deeper insights into whether improvements in job satisfaction, self-efficacy, and professional competencies are sustained over time or diminish without ongoing support. Such studies could also clarify the mechanisms by which job shadowing as a non-formal education programme influences long-term career shifts, further education decisions, and continued professional development (Savickas, 2012; Goodman, 2024).

Finally, future studies should incorporate more robust and objective measures of skill development and career progression, such as supervisor evaluations, employment records, or productivity analytics, to complement self-reported data and reduce potential bias (Joseph and Newman, 2010; Bickerton, 2023). Integrating digital and hybrid formats of job shadowing as a non-formal education programme may also facilitate cross-cultural comparisons and increase accessibility for diverse learner populations.

By pursuing these lines of inquiry, researchers can deepen understanding of the mechanisms and boundary conditions of job shadowing as a non-formal education programme's impact, ultimately informing the design of more effective, inclusive, and sustainable adult education and workforce development programmes.

## 8 Conclusions

This study confirms that integrating social-emotional learning (SEL) principles into job shadowing as a non-formal education programme significantly enhances adult professional development, fostering both practical skill acquisition and emotional growth. The results demonstrate that participation in SEL-integrated job shadowing as a non-formal education programme increases professional confidence, career clarity, and adaptability, thereby supporting adults in a dynamic labor market (Goodman, 2024; Hildmann et al., 2019).

From a theoretical perspective, this research extends Holland's (1997) career theory by demonstrating that SEL functions as a dynamic mediator between personality and environment, transforming static person-environment fit into an adaptive interaction process (Holland, 1997; Joseph and Newman, 2010). The practical findings provide a foundation for the development of modular job shadowing as a non-formal education programme that combines reflective practices with mentor feedback, supporting both technical and emotional competencies (Bickerton, 2023).

For educational policymakers and employers, these conclusions highlight the importance of SEL-integrated job shadowing as a non-formal education programme within lifelong learning frameworks, contributing to workforce resilience and adaptability (Toros, 2025; Goodman, 2024).

Future research directions include:

1. Assessing programme effectiveness across different cultural contexts and industries, especially in technology and healthcare sectors.
2. Incorporating objective performance indicators at the organizational level.
3. Examining long-term impacts on career trajectories and sustained professional growth.

This approach positions job shadowing as a non-formal education programme as a transformative force in adult career development, emphasizing the synergy of skills, emotional intelligence, and social capital for sustainable professional growth (Goodman, 2024; Holland, 1997).

## Data Availability

The raw data supporting the conclusions of this article will be made available by the authors, without undue reservation.
